# The Noradrenergic System in Parkinson’s Disease

**DOI:** 10.3389/fphar.2020.00435

**Published:** 2020-04-08

**Authors:** Elena Paredes-Rodriguez, Sergio Vegas-Suarez, Teresa Morera-Herreras, Philippe De Deurwaerdere, Cristina Miguelez

**Affiliations:** ^1^Department of Pharmacology, Faculty of Medicine and Nursing, University of the Basque Country (UPV/EHU), Leioa, Spain; ^2^Autonomic and Movement Disorders Unit, Neurodegenerative Diseases, Biocruces Health Research Institute, Barakaldo, Spain; ^3^Centre National de la Recherche scientifique, Institut des Neurosciences Cognitives et Intégratives d’Aquitaine (INCIA UMR 5287), Bordeaux, France

**Keywords:** Parkinson’s disease, noradrenaline, locus coeruleus, non-motor symptoms, neuroprotection, prodromic, L-DOPA, neuroinflammation

## Abstract

Nowadays it is well accepted that in Parkinson’s disease (PD), the neurodegenerative process occurs in stages and that damage to other areas precedes the neuronal loss in the substantia nigra pars compacta, which is considered a pathophysiological hallmark of PD. This heterogeneous and progressive neurodegeneration may explain the diverse symptomatology of the disease, including motor and non-motor alterations. In PD, one of the first areas undergoing degeneration is the locus coeruleus (LC). This noradrenergic nucleus provides extensive innervation throughout the brain and plays a fundamental neuromodulator role, participating in stress responses, emotional memory, and control of motor, sensory, and autonomic functions. Early in the disease, LC neurons suffer modifications that can condition the effectiveness of pharmacological treatments, and importantly, can lead to the appearance of common non-motor symptomatology. The noradrenergic system also exerts anti-inflammatory and neuroprotective effect on the dopaminergic degeneration and noradrenergic damage can consequently condition the progress of the disease. From the pharmacological point of view, it is also important to understand how the noradrenergic system performs in PD, since noradrenergic medication is often used in these patients, and drug interactions can take place when combining them with the gold standard drug therapy in PD, L-3,4-dihydroxyphenylalanine (L-DOPA). This review provides an overview about the functional status of the noradrenergic system in PD and its contribution to the efficacy of pharmacological-based treatments. Based on preclinical and clinical publications, a special attention will be dedicated to the most prevalent non-motor symptoms of the disease.

## Introduction

Parkinson’s disease (PD) is a progressive neurodegenerative disorder characterized by the presence of α-synuclein protein aggregates in the form of Lewy bodies in specific brain regions. These aggregates may be responsible for the onset and progression of the disease likely by promoting mitochondrial dysfunction, microglial activation, and neuroinflammatory responses, but they do not appear all over the brain at the same time. Indeed, recent publications have suggested that the α-synuclein pathology begins in the gut and travels *via* the vagal nerve up to the brain where it spreads following the six stages defined by Braak and colleagues (Ulusoy et al., 2013; Kim et al., 2019). Although historically the hallmark of the disease has been focused on the degeneration of the substantia nigra pars compacta (SNc), it is now well accepted that the spread of α-synuclein in the brain occurs in stages and that damage to other areas precedes the degeneration of SNc neurons, affecting glutamatergic, noradrenergic, serotonergic, histaminergic, and cholinergic projection cells (Del Tredici et al., 2002; Braak et al., 2003). This heterogeneous, progressive neurodegeneration may explain the diverse symptomatology of PD, which includes motor and non-motor alterations (Chaudhuri and Schapira, 2009). Indeed, PD is more likely to be a multisystem disorder rather than a pure motor disease.

According to Braak’s theory (Braak et al., 2004), the first α-synuclein aggregates in the central nervous system appear in the anterior olfactory structures and the dorsal motor nucleus of the vagus nerve, following by lower raphe system and the locus coeruleus (LC) in stage 2. It is not until stage 3 that the SNc is affected together with the amygdala, tegmental pedunculopontine nucleus, and the higher raphe nuclei, among others. During stage 4, α-synuclein spreads to the hippocampal formation and specific cortical areas and finally, in the last two stages (5 and 6), almost the whole cortex is damaged. This pattern of α-synuclein propagation between interconnected nuclei has also been mimicked in animal models in which α-synuclein was overexpressed by means of viral vector administration in peripheral structures (Rey et al., 2013; Ulusoy et al., 2013; Ulusoy et al., 2017; Rusconi et al., 2018). The pathological process underlying PD would consist of a prodromal period followed by a symptomatic one when the disease is often diagnosed. The presymptomatic or prodromal phase (stages 1–3) is often characterized by olfactory dysfunction, autonomic dysregulation, pain, sleep, and mood disorders while the symptomatic phase (stages 4–6) is accompanied by the classical somatomotor symptoms and impaired cognitive functioning (Chaudhuri and Schapira, 2009; Braak and Del Tredici, 2016).

Among the brain areas that undergo degeneration in the prodromal phase, the LC deserves special attention for being one of the first nuclei to develop Lewy bodies, and because LC dysfunction may be related to several of the non-motor symptoms observed in the disease. Here, we will review the functional status of the LC in PD using data from experimental models and patients. We will also analyze the potential role of the LC in PD-associated neuroinflammation, the appearance of non-motor complications, and the pharmacological therapies.

## The Locus Coeruleus

The LC is a bilateral nucleus located in the upper dorsolateral pontine tegmentum and is considered the principal noradrenergic nucleus in the central nervous system (Amaral and Sinnamon, 1977). Although noradrenergic neurons are the biggest cell population, GABAergic interneurons also inhabit the LC making synapses and efficiently inhibit the noradrenergic neurons (Aston-Jones et al., 2004; Jin et al., 2016; Breton-Provencher and Sur, 2019). Neurochemical content and receptor expression are also very heterogeneous containing adrenergic, GABAergic, serotonergic, glutamatergic, µ-opioid, orexin/hypocreatin, nicotinic acetylcholine, and cannabinoid receptors (reviewed in Berridge and Waterhouse, 2003; Schwarz and Luo, 2015). LC noradrenergic cells, as happens with SNc neurons, also contains neuromelanin which makes them specially vulnerable to neurodegeneration in PD (reviewed in Martin-Bastida et al., 2017 and Vila, 2019).

Despite being a tiny nucleus, the LC shows an enormous projecting network, influencing the activity of nuclei all over the brain. It sends descending projections to the spinal cord (Westlund et al., 1983) and densely innervates ascending areas of the CNS as the amygdala, superior colliculus, paraventricular thalamic nucleus, hippocampus, olfactory bulb, dorsal raphe, and cortex, including prefrontal, orbitofrontal, anterior cingulate, and primary motor cortices (Fallon et al., 1978; Shipley et al., 1985; Loughlin et al., 1986; Kim et al., 2004; Chandler et al., 2014; Schwarz et al., 2015; Kempadoo et al., 2016; Takeuchi et al., 2016; McCall et al., 2017; Beas et al., 2018; Li L. et al., 2018). The SNc and the ventral tegmental area also receive modest noradrenergic innervation from the LC (Baldo et al., 2003; Mejías-Aponte et al., 2009). By contrast, those areas with intense dopaminergic innervation as the nucleus accumbens or the striatum show discrete noradrenergic innervation (Mason and Fibiger, 1979; Berridge et al., 1997; Delfs et al., 1998; Fitoussi et al., 2013). As for the afferences, the LC also receives a large variety of inputs including those from the paragigantocellularis, prepositus hypoglossi, dorsal raphe, superior colliculus, prefrontal cortex, or the SNc (Aston-Jones et al., 1986; Devoto et al., 2005b; Delaville et al., 2011; Lu et al., 2012; Breton-Provencher and Sur, 2019).

It is interesting to mention that although the LC does not project to nuclei highly innervated by the dopaminergic system, it can still influence dopaminergic transmission distally. Devoto and collaborators have extensively characterized that LC-tyrosine hydroxylase positive fibers can co-release not only noradrenaline (NA) but also dopamine (DA) in the cortex, including prefrontal, parietal, and occipital cortices involving α_2_-adrenoceptor-mediated mechanisms (Devoto et al., 2001; Devoto et al., 2003; Devoto et al., 2004; Devoto et al., 2005a; Devoto et al., 2005b). More recently, other authors have also supported that LC activation promotes DA release in the thalamus and hippocampus, contributing to stress and cognitive functions (Smith and Greene, 2012; Kempadoo et al., 2016; Yamasaki and Takeuchi, 2017; Beas et al., 2018).

In view of the dense noradrenergic projection network, it is easy to understand the implication of this nucleus in many physiological functions and pathological conditions. Experimental preclinical models have demonstrated the implication of the LC in arousal, cognition, anxiety, depression, pain, attention, and locomotor control (Aston-Jones and Bloom, 1981; Carter et al., 2010; Curtis et al., 2012; Sara and Bouret, 2012; Chandler et al., 2014; McCall et al., 2015; Szot et al., 2016; Benarroch, 2017; Hirschberg et al., 2017; McCall et al., 2017; Beas et al., 2018; Breton-Provencher and Sur, 2019; Llorca-Torralba et al., 2019). The availability of new technologies, as opto- and chemogenetics, that allow efficient activation/inhibition of specific anatomical projections or cellular subtypes, has provided a better understanding of those functions and unraveled that the LC is a more heterogeneous nucleus than previously proposed. Interestingly many of the pathological situations triggered by the dysfunction of the LC are present in PD, stressing the role of this nucleus in the development and management of the non-motor complications of the disease.

## Noradrenergic Dysfunction in Parkinson’s Disease

### Preclinical Evidence

In the last decades, researchers have shown increasing interest in further understanding the pathophysiological basis of the non-motor symptoms present in PD with special focus on the noradrenergic system. In the preclinical studies, viral-vector-induced, gene-mutated, or neurotoxin-based animal models are regularly used although the vast majority of the data come from these latter ones.

Anatomical studies using the unilaterally 6-hydroxydopamine (6-OHDA)-lesioned rat model show that the number of LC neurons is not affected by the DA loss (Miguelez et al., 2011b; Ostock et al., 2018) but NA levels in different projection areas are variably decreased. In the prefrontal cortex of the lesioned hemisphere, some authors found unchanged (Delaville et al., 2012a; Delaville et al., 2012b) or reduced NA concentrations (Shin et al., 2014; Ostock et al., 2018). Similarly, other areas with sparse noradrenergic innervation, as the striatum, show unchanged or lower NA levels (Shin et al., 2014; Ostock et al., 2018). Bilateral models of 6-OHDA show, however, more robust NA deficits in the cortex and striatum (Vieira et al., 2019). 1-Methyl-4-phenyl-1,2,3,6-tetrahydropyridine (MPTP)-treated monkeys exhibit clear noradrenergic damage, including LC cell loss, lower NA concentrations in several brain regions, and reduced noradrenergic innervation of the SNc and the subthalamic nucleus (Pifl et al., 1991; Masilamoni et al., 2017). Some publications have also reported low NA striatal and cortical tissue content in MPTP mice (Luchtman et al., 2009; Nayyar et al., 2009; Ando et al., 2018). Indeed, MPTP reproduces better than 6-OHDA the heterogeneous neuronal damage produced in PD, as recently shown by a publication using matrix-assisted laser desorption-ionization mass spectrometry (Kadar et al., 2014). Evidence regarding the integrity of the noradrenergic system in transgenic mice models of PD is more scarce but also stresses noradrenergic impairment. Thus, reduced tyrosine hydroxylase positive cells and α-synuclein aggregations in the LC have been demonstrated in PINK1 knockout rats (Grant et al., 2015; Cullen et al., 2018), LRRK, and parkin knockout mice (Von Coelln et al., 2004; Giaime et al., 2017). However, in transgenic mice overexpressing human A53T α-synuclein, although having lower NA levels at the level of the striatum, olfactory bulb, and spinal cord, TH positive cells in the LC expressed modest α-synuclein aggregates but remained intact in number (Giasson et al., 2002; Sotiriou et al., 2010). These noradrenergic dysfunctions are accompanied by behavioral deficits, such as early vocalization and swallowing deficits.

It is also interesting to stress that in parkinsonian conditions, the noradrenergic system may contribute in some extent to the loss of dopaminergic function. In animals lesioned with 6-OHDA, noradrenergic transporter (NET) are increased possibly for compensating for the severe DA loss (Chotibut et al., 2012). Indeed, NET can reuptake not only NA but also DA in those regions with sparse DA innervation (Morón et al., 2002). In this line, some publications support that in absence of DA transporters, NET reuptakes L-DOPA-derived DA in the striatum and other areas, playing a possible role in L-DOPA induced dyskinesia (LID), as later discussed.

Apart from the anatomical and neurochemical changes, few electrophysiological studies using anaesthetized 6-OHDA lesioned animals, have revealed that experimental DA degeneration also impacts LC neuron basal activity and its response to antidepressant agents in parkinsonian rodents. Regarding the electrophysiological changes, increased and decreased activity was reported by different authors (Wang et al., 2009; Miguelez et al., 2011a; Miguelez et al., 2011b). This discrepancy may rely in methodological differences that could imply direct noradrenergic damage produced by the neurotoxins (Szot et al., 2016). On the other hand, in control rats, serotonergic and noradrenergic antidepressants interact with L-DOPA when administered together (see below). Using the forced swimming test, control rats subchronically treated with L-DOPA and fluoxetine showed reduced efficacy of the antidepressant drug, while coadministration of the NET blocker reboxetine and L-DOPA provided the opposite effect (Miguelez et al., 2013). At the behavioral level, regardless inconsistencies found through the scientific publications, parkinsonian animals tend to mimic the human symptomatology showing motor but also non-motor impairments (Titova et al., 2017). An array of studies report that rodents lesioned with 6-OHDA or MPTP show anxious and depressive behavior, pain, cognitive, and sleep disturbances (Monaca et al., 2004; Pérez et al., 2009; Berghauzen-Maciejewska et al., 2014; Vo et al., 2014; Kamińska et al., 2017; Charles et al., 2018; Campos et al., 2019; Domenici et al., 2019), more notably in bilateral models of the disease (Ferro et al., 2005; Tadaiesky et al., 2008; Santiago et al., 2010; Bonito-Oliva et al., 2014; Vieira et al., 2019). Although the participation of other nuclei cannot be ruled out, the role of the LC in the mentioned functions is widely accepted.

Although preclinical data to large extent support the noradrenergic affection in parkinsonian models, several discrepancies exist, which may be due to methodological variations. In this regard, especially when using the neurotoxin 6-OHDA, it should be considered that lesion protocols vary considerably among the studies, including key steps like animal age, toxin dose and injection site, magnitude of the lesions, and administration of the non-selective NET blocker desipramine to protect noradrenergic neurons from the 6-OHDA toxicity. Genetic and α-synuclein based models are less often used with considerable results variation among the studies. The use of other neurotoxins as MPTP seems to provide results that are more consistent.

### Clinical Evidence

In line with the aforementioned findings reported in animal models of PD, there is substantial evidence showing degeneration of the noradrenergic system in PD patients. Numerous anatomical *post mortem* studies in PD brains have documented a moderate to severe cell loss (around 30–90%) and Lewy body pathology in the LC, equal in magnitude throughout the rostral-caudal parts of the nucleus (Gaspar and Gray, 1984; Chan-Palay and Asan, 1989; German et al., 1992; Bertrand et al., 1997; Zarow et al., 2003; McMillan et al., 2011). Specifically, neuromelanin-containing medium-size LC neurons present somatic and dendritic alterations, whereas smaller non-noradrenergic LC cells do not show severe pathological changes (Patt and Gerhard, 1993).

Although *in vivo* positron emission tomography imaging studies using non-specific ligands failed to identify the noradrenergic damage, more recent neuromelanin-sensitive magnetic resonance studies have found progressive loss of the LC signal in both idiopathic and genetic PD patients and even a lower signal in those PD patients with depressive symptoms (Sasaki et al., 2006; Castellanos et al., 2015; Schwarz et al., 2017; Wang et al., 2018). ^18^F-dopa positron emission tomography imaging, as an index of monoaminergic nerve terminal function, have also demonstrated a reduced uptake in the LC, indicating progressive loss of noradrenergic terminal function (Pavese et al., 2011).

While the relationship between α-synuclein accumulation and neuronal death is not fully understood, it has been proposed that this protein burden may lead to neuronal dysfunction/degeneration and, therefore, impair neurotransmission (Espay et al., 2014). As explained before, Braak and colleagues established six levels of degeneration over the course of the disease where the noradrenergic impairment would occur earlier than the dopaminergic one and the subsequent primary motor symptoms. It has further been proposed that α-synuclein pathology in the LC not only precedes, but may also be of greater magnitude than that occurring in the SNc, a finding that persists across disease stages (Zarow et al., 2003). These results suggest that LC dysfunction may directly contribute to disease onset and progression rather than be a collateral consequence.

Consistent with LC neuron loss and degeneration, there is a decreased noradrenergic innervation of LC target structures, including the prefrontal and motor cortex, striatum, thalamus, hypothalamus, and cerebellum (Kish et al., 1984; Shannak et al., 1994; Pavese et al., 2011; Pifl et al., 2012; Sommerauer et al., 2018b). The atrophy of tyrosine hydroxylase-containing axons is not restricted to the central nervous system, and a prominent loss of noradrenergic innervations of the peripheral autonomic system has been demonstrated, including in the left cardiac ventricle (Hakusui et al., 1994; Takatsu et al., 2000; Slaets et al., 2015).

Although plasma NA levels are elevated in *de novo* PD patients (Ahlskog et al., 1996), neurochemical studies have reported lower levels of the neuronal NA metabolite dihydroxyphenylglycol in the cerebrospinal fluid (Goldstein et al., 2012), as well as marked reduction of DA-beta-hydroxylase activity, an enzyme responsible for hydroxylation of DA to NA, in parkinsonian patients (Hurst et al., 1985; O’Connor et al., 1994). Regarding changes in adrenergic receptors in PD, an *in vitro* autoradiographic study showed upregulation of α_1_- and β_1_- and reduced density of α_2_-adrenoceptors in the prefrontal cortex of *post mortem* parkinsonian patients (Cash et al., 1984).

Despite some discrepancies in animal models, both preclinical and clinical studies support the notion that the noradrenergic system is impaired in parkinsonism. This is important for understanding the complexity of the neurodegenerative process and should be taken into account when administering drugs whose pharmacological effect relies on the integrity of this system.

## Clinical Implications of Noradrenergic Dysfunction in PD

The degeneration of the noradrenergic system in the CNS and periphery occurring in PD is associated with a broad spectrum of non-motor symptoms that encompass autonomic, behavioral, and cognitive parameters. The appearance of these symptoms and signs cannot be just attributed to an alteration in the functioning of the noradrenergic system, as they are also known to be associated with deficits in other neurotransmission systems such as cholinergic, serotonergic, GABAergic, or glutamatergic (Schapira et al., 2017). However, in this review, we will focus on non-motor complications appearing in the prodromal phase of the disease that, apart from other neurotransmitters’ abnormalities, implicate malfunctioning of LC neurons.

In accordance with predicted Braak’s stages, the different clinical features due to noradrenergic dysfunction can be observed along the progression of the disease (Halliday et al., 2011) and, often appear before motor symptoms onset. Detecting noradrenergic impairment could be used as a diagnostic biomarker for early detection of the neurodegeneration, providing an opportunity for intervention with disease-modifying therapies (Betts et al., 2019).

### Autonomic Disturbances

Sympathetic autonomic dysfunction is a common clinical feature of PD and may precede motor symptomatology, becoming more prevalent as the disease progresses (Schapira et al., 2017). The most common dysautonomic symptoms are orthostatic hypotension, urogenital dysfunction, and constipation (Martinez-Martin et al., 2015), but PD patients can also suffer fatigue, thermoregulatory dysregulation, excessive perspiration, or postural light-headedness. Autonomic dysfunction has a heterogeneous manifestation and its progression is not predictable, however, it is associated with reduced autonomy and a decline in quality of life, regardless of severity or duration of the disease (Leclair-Visonneau et al., 2018). The orthostatic hypotension affects 30–58% of PD patients (Goldstein, 2006) and has been linked to peripheral sympathetic cardiovascular denervation and also in a certain degree to central autonomic involvement. PD patients with defined symptomatic orthostatic hypotension exhibit decreased LC neuromelanin signal on magnetic resonance studies (Sommerauer et al., 2018a) and low plasma levels of NA, which is associated with both supersensitivity of vascular adrenergic receptors and an up-regulation of platelet α_2_-adrenoceptors (Senard et al., 1990). As a consequence of a reduced capacity to adapt the peripheral vasculature and cerebral perfusion pressure PD patients can often manifest postural light-headedness and syncope (Sharabi et al., 2008). Constipation and prolonged gastrointestinal transit time affect more than 80% of PD patients and, in some cases, may lead to megacolon (for review see Cersosimo and Benarroch, 2008). Although defecation dysfunction seems to be multifactorial, one of the proposed pathophysiological mechanisms is the accumulation of α-synuclein immunoreactive Lewy bodies in sympathetic ganglia (Wakabayashi and Takahashi, 1997). Urinary and sexual dysfunctions are also late features of PD related to degeneration of brain regions that innervate the bladder, among them the LC (Micieli et al., 2003; Park and Stacy, 2009).

### Sleep Disorders

Sleep disturbances are among the most frequent PD symptoms, affecting some 60–98% of patients (Stacy, 2002). Common sleep disorders include excessive daytime somnolence, nocturnal wakefulness, sleep attacks, REM sleep behavior disorder, or restless leg syndrome. All these sleep impairments can be early premotor manifestations and related to LC dysfunction as underlying mechanism, since this nucleus contributes to the control of arousal and sleep-wake cycle (Carter et al., 2010). In fact, *post mortem* examinations of patients with REM sleep behavior disorder without motor symptoms revealed neuronal loss and Lewy bodies in the LC (Uchiyama et al., 1995). A recent magnetic resonance study further linked LC neuromelanin levels with amount of REM sleep without atonia in PD patients (Sommerauer et al., 2018a). Clinical management of sleep disorders in PD is complex because most of the antiparkinsonian drugs can alter sleep architecture and induce sleepiness as a side effect. Nevertheless clonazepam or melatonin are often prescribed (Gagnon et al., 2006).

### Depression

Depression affects up to 40% of PD patients and may precede the onset of motor symptomatology (Cummings, 1992; Shiba et al., 2000; Cummings et al., 2019). There is strong evidence for a correlation between noradrenergic function and depression in PD patients. In fact, neuroimaging and neuropathological studies have demonstrated reduced LC projections to limbic brain areas (cingulate cortex, thalamus, ventral striatum, or amygdala), as well as gliosis and cell loss at LC level, which was more pronounced in patients with higher frequency of depression or anxiety (Remy et al., 2005; Frisina et al., 2009). As in major depression, selective serotonin reuptake inhibitors, tricyclic antidepressants, serotonin and NA reuptake inhibitors, and monoamine oxidase inhibitors are used in the pharmacotherapy, with partial effect, probably influenced by the neurodegeneration of the serotonergic and noradrenergic systems (Ryan et al., 2019). The use of antidepressant that do not target monoaminergic systems may potentially offer benefit in these patients (Vanle et al., 2018).

### Cognitive Manifestations

The loss of LC neurons and decreased noradrenergic innervation of forebrain targets are associated with cognitive dysfunction in PD (Cash et al., 1987; Rommelfanger and Weinshenker, 2007; Vazey and Aston-Jones, 2012; Sommerauer et al., 2018a). In early PD, subtle cognitive deficits include difficulty in executing functioning, particularly cognitive flexibility, which is the capacity to update and redirect attention when the environmental or homeostatic conditions change. Flexibility in cognitive processing is an essential function of prefrontal cortex and it has been proposed that loss of prefrontal noradrenergic input may contribute to this prodromal cognitive deficit (Vazey and Aston-Jones, 2012). In late stage PD, dementia can occur with a prevalence of 24–31% (Aarsland et al., 2005). Although dementia in PD is related to a substantial reduction in cortical cholinergic markers, there is also evidence for a more severe loss of noradrenergic input from the LC to cortical areas (Chan-Palay and Asan, 1989). The severity of dementia has been linked to the loss of LC neurons in some studies (Zweig et al., 1993; Del Tredici and Braak, 2013; Li et al., 2019).

## Noradrenaline, Neuroinflammation, and Neuroprotection

It is important to mention that NA might protect DA neurons from damage and therefore, integrity of the noradrenergic system may condition the progression of the disease. In this sense, preclinical data from MPTP mice and marmosets suggest that damaging the LC leads to a loss of DA neurons in the SNc followed by more pronounced motor deficits (Mavridis et al., 1991; Marien et al., 1993; Bing et al., 1994; Fornai et al., 1995; Fornai et al., 1997; Yao et al., 2015; Li Y. et al., 2018). Conversely, the damage produced by MPTP is reduced when the synthesis of NA is boosted (Kilbourn et al., 1998; Archer, 2016) or the NET is knocked out (Rommelfanger et al., 2004). A recent publication using mutant mice characterized by the progressive degeneration of dopaminergic neurons demonstrated that chronic pharmacological NET blockade ameliorates such degeneration and the subsequent motor impairment (Kreiner et al., 2019). Peripheral administration of the NET blocker atomoxetine also reduced DA damage in a lipopolysaccharide inflammatory rat model of PD (Yssel et al., 2018). Direct noradrenergic damage by the administration of the neurotoxin N-(2-chloroethyl)-N-ethyl-2-bromobenzylamine (or DSP-4) also produces motor deficits and DA cell loss in control rats (Af Bjerkén et al., 2019). In another recent publication, DSP-4 developed motor and non-motor symptoms in control mice and exacerbated motor disability in mice rendered parkinsonian by the injection of lipopolysaccharide (Song et al., 2019). Some studies performed in 6-OHDA lesioned rodents also suggest that noradrenergic lesions in parkinsonian rats augment dopaminergic neuron vulnerability (Ostock et al., 2014) leading to lower DA levels (Srinivasan and Schmidt, 2003) and worsening motor performance (Srinivasan and Schmidt, 2003; Srinivasan and Schmidt, 2004; Wang et al., 2010; Shin et al., 2014; Ostock et al., 2018). Other studies using the same 6-OHDA model failed, however, to reproduce the latter findings (Delaville et al., 2012a; Guimarães et al., 2013; Ostock et al., 2014; Shin et al., 2014).

The mechanism underlying the neuroprotective effect of NA on DA degeneration is not well understood, although several lines of evidence point at the anti-inflammatory properties of NA as one factor responsible of such an effect. Both PD patients and animal models of the disease show reactive astrogliosis, astrocytic dysfunction, and microglial activation, which has been proposed as the origin of neuroinflammation (Ouchi et al., 2005; Gerhard et al., 2006; Glass et al., 2010; Terada et al., 2016; Liu et al., 2017; Tsutsumi et al., 2019). Importantly, by controlling microglial activation NA is able to halt the damage of dopaminergic neurons. Studies performed in cell cultures and animal models suggest that NA suppresses neuroinflammation by acting, at least in part, on b_2_-adrenergic receptors, which are highly expressed in glial cells (Mori et al., 2002; Tanaka et al., 2002; Yao et al., 2015). Low concentrations of NA or long acting β_2_-agonists are able to inhibit the microglial production and release of chemokines, interleucines, tumor necrosis factor (TNF-α), superoxide or nitric oxide, among others, (Mori et al., 2002; McNamee et al., 2010a; Qian et al., 2011) or to stimulate the synthesis of interleukin-1 receptor antagonists (McNamee et al., 2010b). Pharmaceutical strategies for increasing NA levels also attenuate nigral microglial activation and ameliorate the behavioral deficits in parkinsonian rats (Yssel et al., 2018). Other authors have also proposed that in addition to the β_2_-mediated mechanisms, NA can impact inflammation by inhibiting NADPH oxidase-generated superoxide (Jiang et al., 2015). Additionally, β_2_-agonists and NET inhibitors can also induce release of neurotrophic factors from astrocytes promoting neuroprotection and regeneration (Yssel et al., 2018).

By contrast, NA deficit accelerates dopaminergic neurodegeneration by promoting inflammation, diminishing neurotrophic factors and promoting oxidation in the SN (Yao et al., 2015; Af Bjerkén et al., 2019). In this sense, a recent publication observed that additional lesion of the LC in animal models of PD promotes enhanced release of interleukins and cytokines, likely due to an incorrect microglial function, and aggravates DA neuron degeneration (Yao et al., 2015) although some authors fails to confirm this cell loss (Iravani et al., 2014). As commented before, combined dual NA/DA lesions lead to more severe phenotype including motor and non-motor symptomatology, probably due to the higher inflammatory response and faster cell loss (Bharani et al., 2017; Song et al., 2019). The implication of NA in neuroinflamation and neuroprotection could have an important translational impact in the clinic. In this sense, a long-term prospective observatory study has reported that those patients with chronic respiratory diseases, that are chronically treated with β_2_-adrenergic agonists may have a lower probability of developing PD (Mittal et al., 2017). One of the reasons for this neuroprotective effect may not only rely on microglial activation, but also on the ability of b_2_-adrenoceptors to down regulate expression of human α-synuclein genes and moderate protein expression. When looking for new neuroprotective therapies, it may be relevant to assure a good NA tone for minimizing the contribution of neuroinflammation to the neuropathology.

## L-DOPA and the Noradrenergic System

L-DOPA is still considered the most efficient anti-parkinsonian drug. It is a metabolic precursor of NA through its decarboxylation into DA by the aromatic amino acid decarboxylase and the β-hydroxylation of DA by the DA beta-hydroxylase. Unfortunately, L-DOPA is poorly effective against non-motor symptoms, does not control some later-onset motor problems like freezing or “wearing-off” fluctuations, and its long-term use is associated with dyskinesia and hallucinations (Olanow et al., 2009; Hirao et al., 2015). The contribution of the noradrenergic system to LID has been investigated in animal models using NA/DA neurotoxic lesions. Although some studies report no implication of the noradrenergic system (Pérez et al., 2009; Ostock et al., 2014; Ostock et al., 2018), others have demonstrated that additional noradrenergic lesion worsens dyskinetic movements in parkinsonian rodents chronically treated with L-DOPA (Fulceri et al., 2007; Miguelez et al., 2011b; Shin et al., 2014). Interestingly, in one study, the authors induced the noradrenergic lesion to already dyskinetic animals, showing an increase in the duration of the dyskinetic effect of L-DOPA probably due to impaired striatal DA clearance (Miguelez et al., 2011b).

Some noradrenergic drugs have proven antidyskinetic properties in experimental animal models of LID. The α_2A_-adrenoceptor antagonist idazoxan, reduced LID, and delayed their onset without compromising the motor score in MPTP-treated monkeys. Idazoxan prevented LID appearance while increasing the locomotor response to L-DOPA (Henry et al., 1999; Grondin et al., 2000; Fox et al., 2001). Additional α_2A_-adrenoceptor antagonists have proven similar antidyskinetic properties (Gomez-Mancilla and Bédard, 1993; Henry et al., 1999; Grondin et al., 2000; Savola et al., 2003; Fox and Brotchie, 2010). Other noradrenergic drugs, as the β-adrenergic receptor antagonist propranolol and the α_2_-receptor agonist clonidine also showed antidyskinetic effects in MPTP-treated monkeys, at the cost of reducing the antiparkinsonian efficacy (Gomez-Mancilla and Bédard, 1993). Experiments performed in 6-OHDA lesioned rats support the prodyskinetic action of the α_2_-antagonist atipamezole and the antidyskinetic effect of propranonol, clonidine, or idazoxan, which did not worsen motor performance (Dekundy et al., 2007; Gerlach et al., 2013; Bhide et al., 2015; Ostock et al., 2015). These findings suggest that the limited though beneficial effect of clonidine on LID is probably indirect due to the stimulation of somatodendritic α_2_-receptors, which inhibit noradrenergic neuronal activity and NA release. Some authors also suggest that the antidyskinetic effect of clonidine may be related to its sedative properties (Gerlach et al., 2013). Conversely, the antidyskinetic action of idazoxan, even if it enhances brain NA release by antagonizing α_2_ autoreceptors, could be related to the blockade of α_2_-receptors expressed by NA receptive cells at terminal levels. In any case, the overall picture is complex due to the involvement of the other adrenergic receptor subtypes in LID. The resulting effect of an overall increase in NA extracellular on LID is thus uncertain. In contrast to the beneficial effect of reboxetine (Shin et al., 2014), other NET inhibitors including desipramine have been reported to aggravate LID (Arai et al., 2008; Chotibut et al., 2014; Conti et al., 2016).

As mentioned above, noradrenergic mechanisms are involved in non-motor symptoms and shape the responses to other medications such as antidepressant drugs (Eskow Jaunarajs et al., 2010; Eskow Jaunarajs et al., 2011; Miguelez et al., 2011b; Miguelez et al., 2013). Noradrenergic neurons may also be involved in the effects of L-DOPA, but the preclinical data are not clear. For instance, participation of noradrenergic neurons in the ability of L-DOPA to enhance DA extracellular levels is likely an indirect effect that requires the dorsal raphe nucleus (Tanaka et al., 1999; Navailles et al., 2010; Navailles and De Deurwaerdère, 2012; De Deurwaerdère et al., 2017; Miguelez et al., 2017). In dyskinetic rats, lesion of noradrenergic terminals or neurons did not reduce L-DOPA-stimulated extracellular levels of DA in the striatum (Navailles et al., 2014; Ostock et al., 2018). However, the noradrenergic lesion enhanced the effect of L-DOPA in extrastriatal regions in part due to the loss of clearance of extracellular DA by noradrenergic fibers bearing NET (Navailles et al., 2014). Another study found that the noradrenergic lesion with DSP-4 potentiates L-DOPA-induced rotations, although this behavioral effect is not directly related to DA extracellular levels (Pérez et al., 2007). Evidence suggests that the noradrenergic system participates in the effect of L-DOPA, but preclinical data are difficult to extrapolate to PD patients because the noradrenergic fibers, at least from the LC, are damaged (see above). It is also important to bear in mind that extracellular striatal DA levels induced by L-DOPA in the striatum neither parallel motor effects nor abnormal motor effects (De Deurwaerdère et al., 2017).

Although behavioral data support that noradrenergic drugs can modulate the effect of L-DOPA, the potential effect of L-DOPA on NA content or neuron activity is less clear. In the LC, low doses of L-DOPA did not alter the electrical tonic activity of noradrenergic neurons in control rats (Miguelez et al., 2013). *Post mortem* data vary, reporting no effect, or a decrease in NA tissue concentration in response to L-DOPA administration depending on the dose regimen and the brain region studied (for review see De Deurwaerdère et al., 2017). Acute or chronic L-DOPA increased NA tissue level in the prefrontal cortex of normal macaques, but the same regimen decreased NA tissue level in the prefrontal cortex and the amygdala of MPTP-treated monkeys whether they were dyskinetic or not (Engeln et al., 2015). The only difference regarding NA tissue levels between non-dyskinetic and dyskinetic monkeys was found at the level of the motor cortex. Studies on L-DOPA-evoked extracellular levels of NA also show inconclusive results. In this regard, the latest publications have reported a substantial increase in striatal NA release after L-DOPA administration (Wang et al., 2014; Ostock et al., 2018). However, in one study NA levels were still excessive after noradrenergic neurons were destroyed (Ostock et al., 2018). It is likely that other electrochemically active compounds were confounding the chromatograms (Chagraoui et al., 2019). Other data indicate that L-DOPA either inhibits or does not alter NA release in the cortex (Dayan and Finberg, 2003; Pascucci et al., 2012).

## Conclusion

The identification of noradrenergic mechanisms in PD is crucial for understanding autonomic functions and non-motor symptomatology, and drugs that target this system may have a beneficial impact in the quality of life of the patients. One major difficulty is to extrapolate the results from animal models to patients where those alterations are variable and depend on the stage of the disease. Meanwhile, the involvement of the noradrenergic system in L-DOPA induced therapeutic effects is controversial, and noradrenergic strategies to limit the side effects accompanying anti-parkinsonian drugs are still not firmly established. In summary, and taking into account that noradrenergic system pathophysiology is a common feature of PD with other neurodegenerative diseases, such as Alzheimer’s disease or atypical neurodegenerative dementias, maintenance of this system integrity may provide a common viable therapeutic option as neurodegenerative diseases-modifying strategy.

## Author Contributions

All authors contributed to writing the manuscript and approved the final version.

## Funding

This study was supported by grants from the Basque Government (PIBA 2019-38, IT1345-19), UPV/EHU (PPGA19/15), and Spanish Government (SAF2016‐77758‐R [AEI/FEDER, UE]). EP-R has a fellowship from the Basque Country and SV-S from the UPV/EHU.

## Conflict of Interest

The authors declare that the research was conducted in the absence of any commercial or financial relationships that could be construed as a potential conflict of interest.
